# Effect of drought stress on the genetic architecture of photosynthate allocation and remobilization in pods of common bean (*Phaseolus vulgaris* L.), a key species for food security

**DOI:** 10.1186/s12870-019-1774-2

**Published:** 2019-04-30

**Authors:** Jorge C. Berny Mier y Teran, Enéas R. Konzen, Antonia Palkovic, Siu M. Tsai, Idupulapati M. Rao, Stephen Beebe, Paul Gepts

**Affiliations:** 1 Department of Plant Sciences, University of California, Davis, CA USA; 2 Cell and Molecular Biology Laboratory, Centro de Energia Nuclear na Agricultura (CENA), Universidade de São Paulo, Piracicaba, SP Brazil; 3 Present Address: Universidade Federal do Rio Grande do Sul, Campus Litoral Norte, Imbé, RS Brazil; 4 Centro Internacional de Agricultura Tropical (CIAT), Cali, Colombia; 5 United States Department of Agriculture, Plant Polymer Research Unit, National Center for Agricultural Utilization Research, Agricultural Research Service, Peoria, Il USA

**Keywords:** Abiotic stress, Epistasis, Photosynthate remobilization, Pod harvest index, Quantitative trait loci

## Abstract

**Background:**

Common bean is the most important staple grain legume for direct human consumption and nutrition. It complements major sources of carbohydrates, including cereals, root crop, or plantain, as a source of dietary proteins. It is also a significant source of vitamins and minerals like iron and zinc. To fully play its nutritional role, however, its robustness against stresses needs to be strengthened. Foremost among these is drought, which commonly affects its productivity and seed quality. Previous studies have shown that photosynthate remobilization and partitioning is one of the main mechanisms of drought tolerance and overall productivity in common bean.

**Results:**

In this study, we sought to determine the inheritance of pod harvest index (PHI), a measure of the partitioning of pod biomass to seed biomass, relative to that of grain yield. We evaluated a recombinant inbred population of the cross of ICA Bunsi and SXB405, both from the Mesoamerican gene pool, to determine the effects of intermittent and terminal drought stresses on the genetic architecture of photosynthate allocation and remobilization in pods of common bean. The population was grown for two seasons, under well-watered conditions and terminal and intermittent drought stress in one year, and well-watered conditions and terminal drought stress in the second year. There was a significant effect of the water regime and year on all the traits, at both the phenotypic and QTL levels. We found nine QTLs for pod harvest index, including a major (17% of variation explained), stable QTL on linkage group Pv07. We also found eight QTLs for yield, three of which clustered with PHI QTLs, underscoring the importance of photosynthate remobilization in productivity. We also found evidence for substantial epistasis, explaining a considerable part of the variation for yield and PHI.

**Conclusion:**

Our results highlight the genetic relationship between PHI and yield and confirm the role of PHI in selection of both additive and epistatic effects controlling drought tolerance. These results are a key component to strengthen the robustness of common bean against drought stresses.

**Electronic supplementary material:**

The online version of this article (10.1186/s12870-019-1774-2) contains supplementary material, which is available to authorized users.

## Background

Yield potential is genetically determined by the efficiencies in light interception, conversion of light to photosynthates, and translocation and partitioning of photosynthates to biomass of the harvestable organ [1, 2]. For biomass partitioning to seeds, breeding efforts in major crops have achieved significant gains [3]. Nevertheless, partitioning remains relatively low in most pulses [4]. Furthermore, photosynthate partitioning and remobilization are important mechanisms of drought tolerance in common bean [5, 6]. Other mechanisms, like conservative transpiration [7, 8] and deep rooting [9, 10] can help maintain high water status at pod filling, thus enhancing remobilization, increasing the harvest index and grain yield.

Understanding the genetic basis of yield productivity, physiological efficiency (rates of biomass and seed yield accumulation), and drought resistance mechanisms is an essential component for the design and implementation of efficient breeding strategies and marker-assisted selection for drought tolerance [11–13]. The timing and severity of the drought stress can significantly alter the balance between vegetative and reproductive growth [4]. For example, stress during flowering can induce flower drop and, in turn, cause more vigorous, compensatory vegetative growth, while drought stress during pod filling would cause more biomass production and decreased sink strength, resulting in a low harvest index [5]. In common bean, pod walls serve as a temporary storage of carbohydrates before rapid seed growth and efficient translocation of carbohydrates during grain filling. Thus, carbohydrate storage in pod walls and their translocation to seeds is of great importance for productivity [14].

Common bean (*Phaseolus vulgaris* L.; 2n = 2x = 22) is the most important legume for direct human consumption, because of its wide consumption and cultivation, and nutritional and agronomic role, which complements that of cereals and other sources of carbohydrates like root crops and plantain [15, 16]. Its role as a complementary source of nutrition is limited, nevertheless, because more than half of its area of production is affected by drought, which causes yield reduction or even crop failure as the production of common bean is mostly rainfed [17, 18]. Terminal (TD) and intermittent (ID) drought are two of the patterns of drought most common in bean production. The specific growth and development stage affected by drought stress, as well as its duration and intensity, are major factors affecting yield and seed quality [17, 19]. In addition, one of the major effects of global warming is the change of patterns and reliability of rainfall, which will likely increase drought severity, especially near the tropics [20]. An important approach to coping with drought constraints and maintain or increase productivity is through breeding and development of resilient cultivars [21, 22].

Yield, and its components, are quantitative traits under polygenic control [23–25]. Pod Harvest Index (PHI), defined as the partitioning of biomass from pods to seeds, has been identified as a useful and stable trait for indirect yield selection in drought and non-drought stressed environments [19, 26, 27]. Previous efforts in QTL discovery for PHI have identified associated genomic regions in the presence or absence of drought stress [28–30] or only in well-watered conditions [31, 32]. However, any differential expression of PHI QTLs between terminal and intermittent drought has not been investigated to our knowledge. Furthermore, variation in PHI is determined by variation in allocation of photosynthates to pod and seed, as well as the capacity of the remobilization from the pod wall to the seed; these three factors could be differentially controlled genetically. Although the role of epistasis in common bean has been quantified previously for yield components [23] and pod shape [33, 34], there are no estimates of epistasis for PHI and its components. Therefore, the objectives of the research presented here were to: 1) estimate the differential effect of intermittent and terminal drought on yield, PHI, and its components; 2) identify QTLs for these traits; and 3) estimate the role of pleiotropy-tight linkage and epistasis in controlling PHI.

## Results

### Phenotypic segregations

#### Comparison of parents across environments

**Fig. 1:**
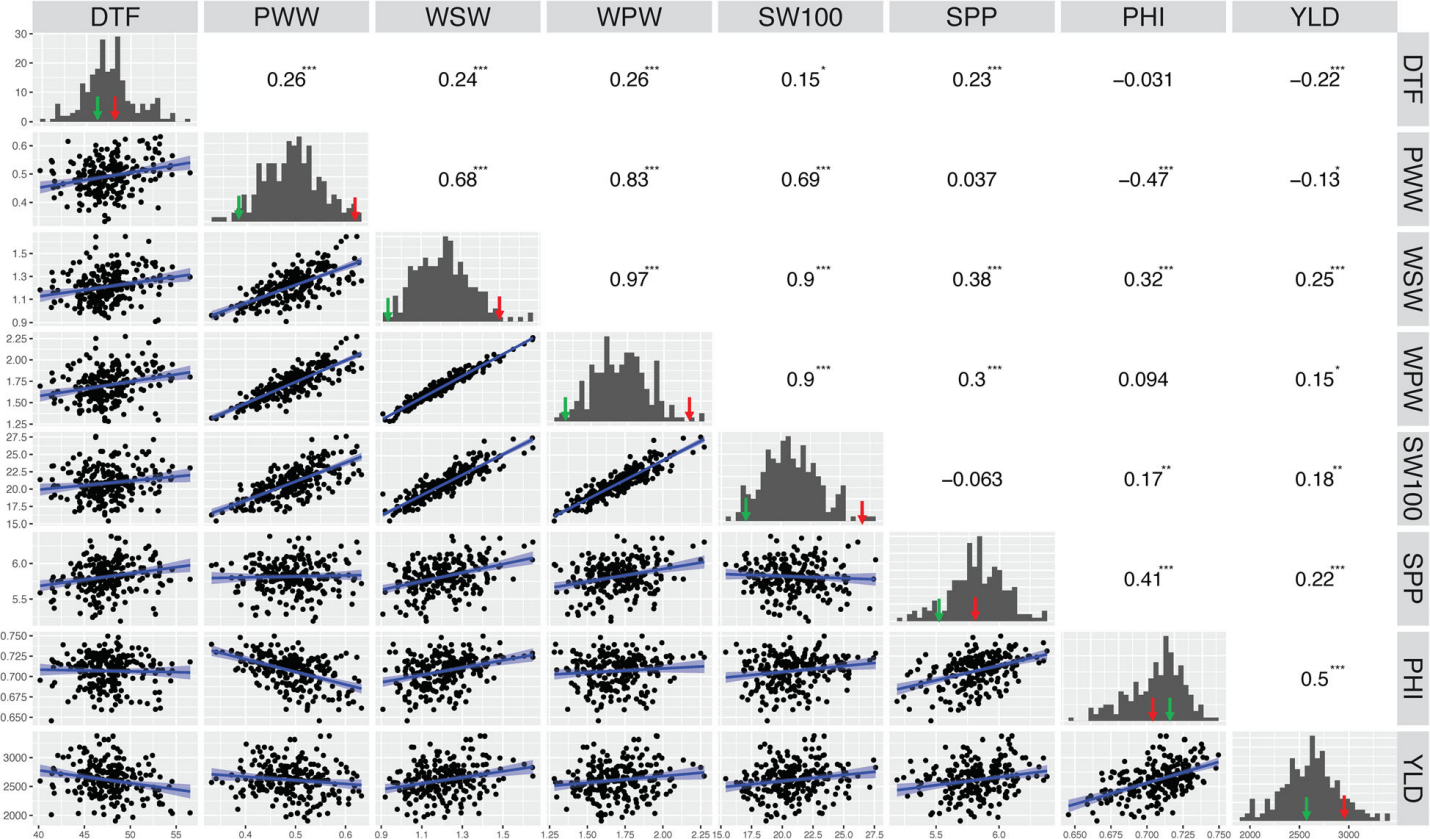
Matrix of trait frequency distribution (diagonal), joint distribution (lower triangle), and correlation coefficient (upper triangle) of the traits averaged across environments in the ICA Bunsi x SXB405 RIL population. Traits: days to flowering (DTF), pod wall weight (PWW), whole seed weight (WSW), whole pod weight (WPW), 100- seed weight (SW100), seeds per pod (SPP), pod harvest index (PHI), Yield (YLD). Parental means are showed as arrows in red (ICA Bunsi) and green (SXB405)

ICA Bunsi and SXB405 differ significantly for pod-wall weight (PWW), whole-seed weight (average seed dry weight per pod: WSW), whole-pod weight (average dry pod weight, including seeds: WPW), 100-seed weight (SW100), but not for days to flowering (DTF), number of seeds per pod (SPP), pod harvest index PHI), and yield (YLD; Table 1). SXB405 had higher phenotypic values for all traits, except for PHI. SXB405 had 61, 59 and 60% higher weight in pod wall, seed and whole pod weight, respectively. It also had a 100-seed weight that was 10 g heavier (59%). In all traits, there was transgressive segregation among the RILs, resulting in phenotypic values that went beyond parental values (Table 1; Fig. 1). For flowering time, some genotypes were six days earlier than ICA Bunsi, and 10 days later than SXB405. For PHI, the highest line showed a value of 0.76, compared with the high parent (0.71). For yield, the highest line had 3682 kg ha^− 1^ across environments, which is 24% higher than SXB405. For PWW, WSW, and WPW, the transgressive segregation was less marked.

**Table 1:** Descriptive statistics and analysis of variance for 8 traits: days to flowering (DTF), pod wall weight (PWW), whole-seed weight (WSW), whole-pod weight (WPW), 100-seed weight (SW100), seeds per pod (SPP), pod harvest index (PHI), and yield (YLD), in three water regimes in two year

	Trait	DTF	PWW	WSW	WPW	SW100	SPP	PHI	YLD
	Units		g pod^−1^	g pod^−1^	g pod^−1^	g 100-seed^− 1^		WSW / WPW	kg ha^− 1^
Parent mean^1^	ICA Bunsi	46.0	0.38A	0.93A	1.31A	16.87A	5.49	0.71	2619
	SXB405	47.8	0.62B	1.48B	2.09B	26.92B	5.54	0.70	2969
RIL	Mean	47.6	0.49	1.22	1.71	20.88	5.87	0.71	2497
	Min	40.9	0.32	0.90	1.22	14.93	4.92	0.64	1445
	Max	57.4	0.65	1.78	2.40	28.57	6.67	0.76	3682
F values^2^	Genotype	35.3***	25.9***	19***	21.5***	36.8***	5.8***	16.1***	8.4***
	Env	114.1***	41.2***	99.5***	82.2***	241.9***	127.4***	174.1***	325.9***
	GxE	1.9***	1.2**	1.7***	1.6***	1.7***	1.6***	1.7***	2.6***
Environmental means^3,4^	2013-C	49.64 A	0.48 C	1.36 A	1.85 A	21.06 C	6.48 A	0.74 A	2515 B
	2013-TD	49.3 B	0.49 B	1.33 B	1.81 B	21.98 A	6.53 A	0.74 AB	1846 C
	2013-ID	49.02 B	0.48 C	1.35 AB	1.84 AB	21.37 B	6.3 B	0.73 C	
	2014-C	45.53 D	0.48 C	1.07 C	1.58 C	19.24 ABCD	4.98 ABC	0.67 C	3173 A
	2014-TD	44.64 C	0.52 A	0.95 D	1.43 D	20.42 D	4.86 C	0.66 D	2456 B
CV		3.2	7.4	8.6	7.5	5.6	6.7	2.4	17.7
R squared		0.81	0.70	0.78	0.76	0.78	0.76	0.80	0.70
Heritability		0.94	0.96	0.92	0.93	0.96	0.73	0.90	0.68

^1^Levels not connected with the same letter are significantly different according to the Student’s t test (*P* < 0.05)

^2^Differences among RILs: significance codes: ‘***’ < 0.001, ‘**’, < 0.01

^3^Levels not connected with the same letter are significantly different according to the Tukey-Kramer HSD test (P < 0.05)

^4^Treatments: full irrigation (C), terminal drought (TD), intermittent drought (ID)

#### Sources of variation

Among RILs, the effects of genotype, environment and their interaction were significant for all traits at the *P* < 0.001 level (Table 1). In general, the differences between the two years were larger than those between treatments. For days to flowering, the effect of year was significant. Within years, the drought treatments caused significantly earlier flowering, although the average difference was less than one day. For PWW, terminal drought led to heavier pod walls in both years compared to the control. For WSW and WPW, reductions were observed in both years compared to the control. For 100-seed weight (SW100), terminal drought led surprisingly to heavier seeds than intermittent drought in the first year, and to the control in the two years. SPP was lower under intermittent drought, compared to the control and terminal drought in the first year. The second year had significantly lower number of seeds per pod, with no statistical difference among treatments. PHI was significantly lower in the well-watered control and only in the intermittent drought in the first year. In the second year, PHI under terminal drought treatment was also lower than in the control treatment. In both comparisons, however, the reduction in PHI was only 0.01. For yield, there was a reduction of 27% under terminal drought in the first year and 23% in the second year, compared to the well-watered control (Table 1).

#### Correlations among traits

PHI (r = 0.5), followed by WSW (r = 0.25) and SPP (r = 0.22) (Fig. 1, Table 2) were the traits most positively correlated with yield. In contrast, DTF was negatively correlated with yield (r = − 0.22). PHI had a higher (negative) correlation with the weight of pods (r = − 0.47) than to the number of seeds (r = 0.032). There was a relatively high positive correlation of the number of seeds per pod and pod harvest index (r = 0.41). SPP was correlated with the whole-pod seed weight (r = 0.38) but not significantly correlated with whole-pod weight. Correlations between yield and yield-associated traits in each year (Table 2), were similarly significant between treatments (fully irrigated control and terminal drought) within each year. WSW, WPW, SW100, SPP, and PHI were significantly correlated with yield in all year/treatment combinations. In contrast, DTF and WPW weight were only significantly (and negatively) correlated with yield in 2014. For DTF, the correlation was higher in the drought treatment than in the full irrigation treatment in both years. The correlation between yield and PHI was similar between treatments but lower in 2013 than in 2014, with 0.28, 0.26, 0.55 and 0.53 for 2013I, 2013D, 2014I and 2014D, respectively.

**Table 2:** Correlation coefficients between grain yield and other traits studied in the ICA-Bunsi x SXB405 RIL population grown under different irrigation schemes and years

Trait	2013 Irrigated	2013 Terminal Drought	2014 Irrigated	2014 Terminal Drought
Days to flowering	0.06	0.09	−0.33***	−0.46***
Pod wall weight	0.12	0.12	−0.17*	−0.13*
Whole seed weight	0.36***	0.36***	0.37***	0.36***
Whole pod weight	0.31***	0.31***	0.23***	0.23***
Seed weight	0.31***	0.25***	0.23***	0.15*
Seeds per pod	0.18***	0.20***	0.32***	0.38***
Pod harvest index	0.28***	0.26***	0.55***	0.53***

Significance codes: ‘***’ < 0.001, ‘*’ < 0.05

### Molecular linkage map and QTL analyses

#### Molecular linkage map

**Fig. 2:**
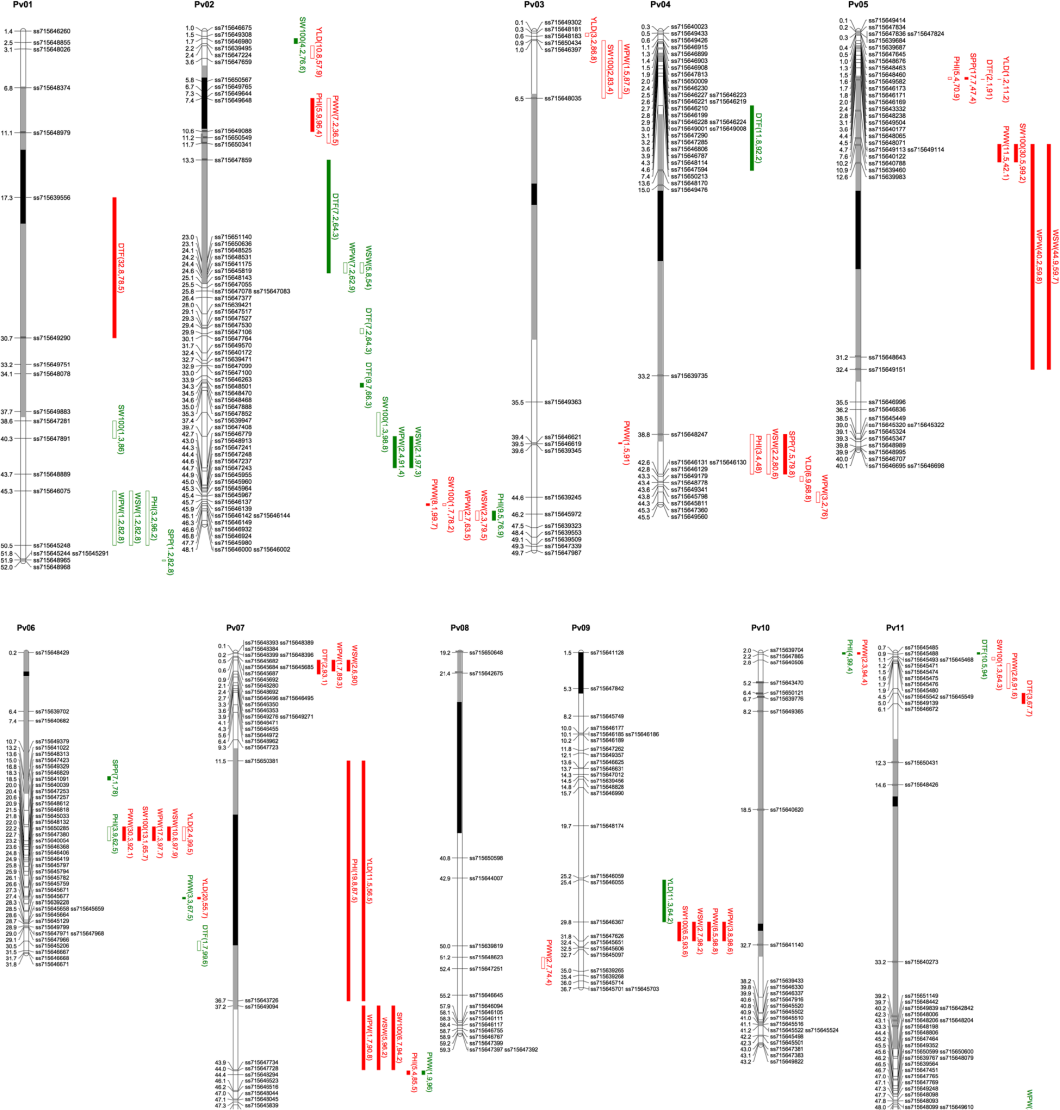
Linkage groups and QTLs of the ICA Bunsi x SXB405 RI population for pod harvest index (PHI), pod wall weight (PWW), whole seed weight (WSW), whole pod weight (WPW), 100-seed weight (SW100), seeds per pod (SPP), days to flowering (DTF) and yield (YLD). Full bars represent QTLs that were detected in all years or in the overall mean across environments. Open bars represent QTLs that were not consistent across environments. The length of the bars represent the 99% confidence interval. The color of the bars represents the positive effect of the parent, ICA Bunsi in green and SXB405 in red. The positions in the linkage group are the physical positions of the markers in Mb and plotted with MapChart [36]

The length of the linkage map was 951 cM using 378 markers across the 11 linkage groups of the common bean genome (Fig. 2). There were on average 34 markers per linkage group with an average and range of spacing of 3.0 and 1.7–5.3 markers/cM, respectively (Table 3). By comparing the genetic map with the physical map using shared markers with the reference genome of G19833 (Version 1.0; [35]), the map covered a physical length of 479,410,006 base pairs or 82% of the total sequence length of the 587 Mb bean genome. The coverage of the genome among linkage groups ranged from 98 to 67% of physical length. The average maximum interval without markers across linkage groups was 19 cM long. The longest interval was on linkage group Pv03 with 29 cM between flanking markers. A relatively large part of the short arm of linkage group Pv08 (~ 20 Mbp from the beginning of the linkage group) was nearly devoid of polymorphic SNPs. The average physical distance (kbp) per unit of genetic distance (cM) was 516 kbp/cM and ranged between 362 kbp/cM (Linkage group Pv09) and 680 Kbp/cM (Linkage group Pv10).

**Table 3:** SNP distribution among the linkage groups/chromosomes of the ICA Bunsi x SXB405 RIL population

Linkage groups	No. of markers	Length linkage groups	Average spacing	Maximum spacing	Physical range		Physical length	Recombi-nation distance	Genome coverage
		cM	Marker cM^−1^	cM	bp	bp	bp	Kbp cM^−1^	%
Pv01	19	95	5.3	24.5	1,401,251	52,025,956	50,624,705	532.9	97.0
Pv02	64	104.5	1.7	11.8	1,026,678	48,100,260	47,073,582	450.5	96.0
Pv03	17	83.2	5.2	29.3	61,877	49,728,839	49,666,962	597.0	95.0
Pv04	41	101.1	2.5	17	261,173	45,468,422	45,207,249	447.2	98.4
Pv05	41	89.6	2.2	20.7	129,685	40,309,715	40,180,030	448.4	98.4
Pv06	42	77.1	1.9	13	200,825	31,799,267	31,598,442	409.8	98.8
Pv07	40	100.4	2.6	15.3	61,520	50,054,206	49,992,686	497.9	96.6
Pv08	17	59.6	3.7	24.2	19,218,321	59,337,170	40,118,849	673.1	67.2
Pv09	28	97.3	3.6	16.6	1,487,977	36,698,444	35,210,467	361.9	94.0
Pv10	24	60.6	2.6	11.8	1,969,500	43,194,406	41,224,906	680.3	95.3
Pv11	45	82.8	1.9	21.5	730,195	49,242,323	48,512,128	585.9	96.3
Average	34.4	86.5	3.0	18.7	2,413,546	45,996,273	43,582,728	516.8	93.9
Total	378	951.2					479,410,006		

#### Identification of additive QTLs and their distribution on the molecular linkage map

Quantitative trait loci analyses were performed for each treatment and year combination as well as for the overall mean across environments, as an indication of the degree of stability of each QTL (Table 4, Additional file 1: Table S1, and Fig. 2). We found 51, 48, 49, 40, 41, 51 QTLs for the 2013-C, 2013-TD, 2013-ID, 2014-C, 2014-TD environments and overall mean, respectively.

**Table 4:** Number of QTLs and percent of variation explained for the 5 environments and overall performance

Trait	2013-C		2013-TD		2013-ID		2014-C		2014-TD		Mean	
	N	PVE	N	PVE	N	PVE	N	PVE	N	PVE	N	PVE
DTF	8	74.4	8	73.4	8	73.5	8	56.0	7	55.4	8	73.1
PWW	9	63.1	6	48.6	9	64.4	5	50.3	5	50.3	10	67.8
WSW	7	60.7	4	53.7	7	60.0	7	53.4	7	52.9	6	59.2
WPW	7	62.6	9	64.5	8	63.9	7	54.3	6	50.9	8	63.2
SW100	8	63.2	9	67.9	8	64.4	4	40.3	7	59.7	7	62.0
SPP	3	27.0	3	31.2	3	29.3	1	12.1	1	12.1	4	23.6
PHI	6	50.5	6	49.2	6	52.4	3	40.4	3	40.8	4	43.1
YLD	3	17.9	3	21.0			5	46.1	5	41.8	4	33.3

PVE: proportion of value explained, as the linear sum of the maximum values corresponding to each marker

There were QTLs in all linkage groups. Nevertheless, only one QTL was found in linkage groups 08 and 10. Across environments, the percentage of explained variation (PVE) ranged from 24% (SPP) to 73% (DTF) (Table 4). PHI and YLD had a percentage of explained variation of 43 and 33%, respectively. Interestingly, PHI had higher PVE in 2013 than in 2014, while for yield PVE was lower in 2013 and higher in 2014.

For days to flowering, 10 QTLs were found in eight linkage groups. Five of these QTLs were significant across all environments. The remaining QTLs were year-specific, and not treatment-specific. In four of the 10 QTLs, ICA Bunsi contributed the negative allele (earliness). The QTL with the largest percentage of explained phenotypic variation was located on linkage group Pv01 with 26% of the additive variance across environments, an additive effect of 1.2 days, and ICA Bunsi contributing to earliness.

#### Photosynthate allocation and remobilization traits

For PWW, 11 QTLs were found distributed over nine linkage groups. The ICA Bunsi allele contributed positively for only three of them. Four QTLs were expressed in all environments. The QTL explaining the largest variation at 19 cM in Pv06, explaining 29%, with SXB405 allele having the positive effect. Three QTLs were expressed only in 2013, in the control and terminal drought treatments, but not in the intermittent drought treatment. There were 10 QTLs for WSW, distributed on seven linkage groups. SXB405 was the source of the positive allele in six QTLs.

Similar to PWW, the largest QTLs for WPW were located on Pv05 and Pv06, with 28 and 11% of the variance across environments explained, and SXB405 being the source of the positive allele. For WPW, there were 12 QTLs distributed on nine linkage groups. The positive allele was contributed by SXB405 for eight QTLs. Two relatively large QTLs were found on Pv05 and Pv06, with 24 and 17% of the additive variation. In both cases, SXB405 was the source of the positive allele. These two QTLs were the only ones being expressed across environments.

For PHI, there were nine QTLs on seven linkage groups. ICA Bunsi alleles contributed positively to five QTLs. Only the QTL at the 59 cM position on Pv07 was expressed across environments. This QTL also had the highest percentage of the variation explained (17%), with SXB405 contributing the positive allele. In general, the other QTLs were expressed similarly between treatments, but not between the two years. Five QTLs were significant in all treatments in 2014, but not in 2013, while one QTL was significant across treatments in 2013 but not in 2014. The QTL on Pv01 was significant only under terminal drought in 2014. The QTL in Pv04 was significant only in the control conditions in both years.

For SPP, four QTLs were found. All QTLs were expressed in 2013, but only one in 2014. The QTL explaining the largest variation was located on Pv05 (8%), the allele of SXB405 had the positive effect. While the other three QTLs positive effect was from the ICA Bunsi. There were 10 QTLs for SW100 distributed in eight linkage groups. SXB405, the parent with the heaviest seed, was the source of the allele with positive effect in seven QTLs. The QTL on Pv05, had the largest explained variation with 30% of the additive variation, and limited environmental influence. The SXB405 allele increased the seed weight by 1.27 g per hundred seeds over the mean.

#### Yield

For YLD, eight QTLs were found across seven linkage groups. SXB405 was the source of the positive allele in six of these QTLs. None of the QTLs were significant in all environments. The differential expression was mostly due to the year than to the treatment effect; none of the QTLs were significant in both years. In 2014, all the QTLs expressed were significant in both treatments. However, in 2013, two of the three QTLs were significant only under terminal drought. Nevertheless, the analysis across environments detected three QTLs, of which the major QTL in Pv06 explained 11% of the additive variation. The positive allele was conferred by SXB405, increasing yield by 106 kg ha^− 1^ above the average.

#### Clustering of QTLs

**Fig. 3:**
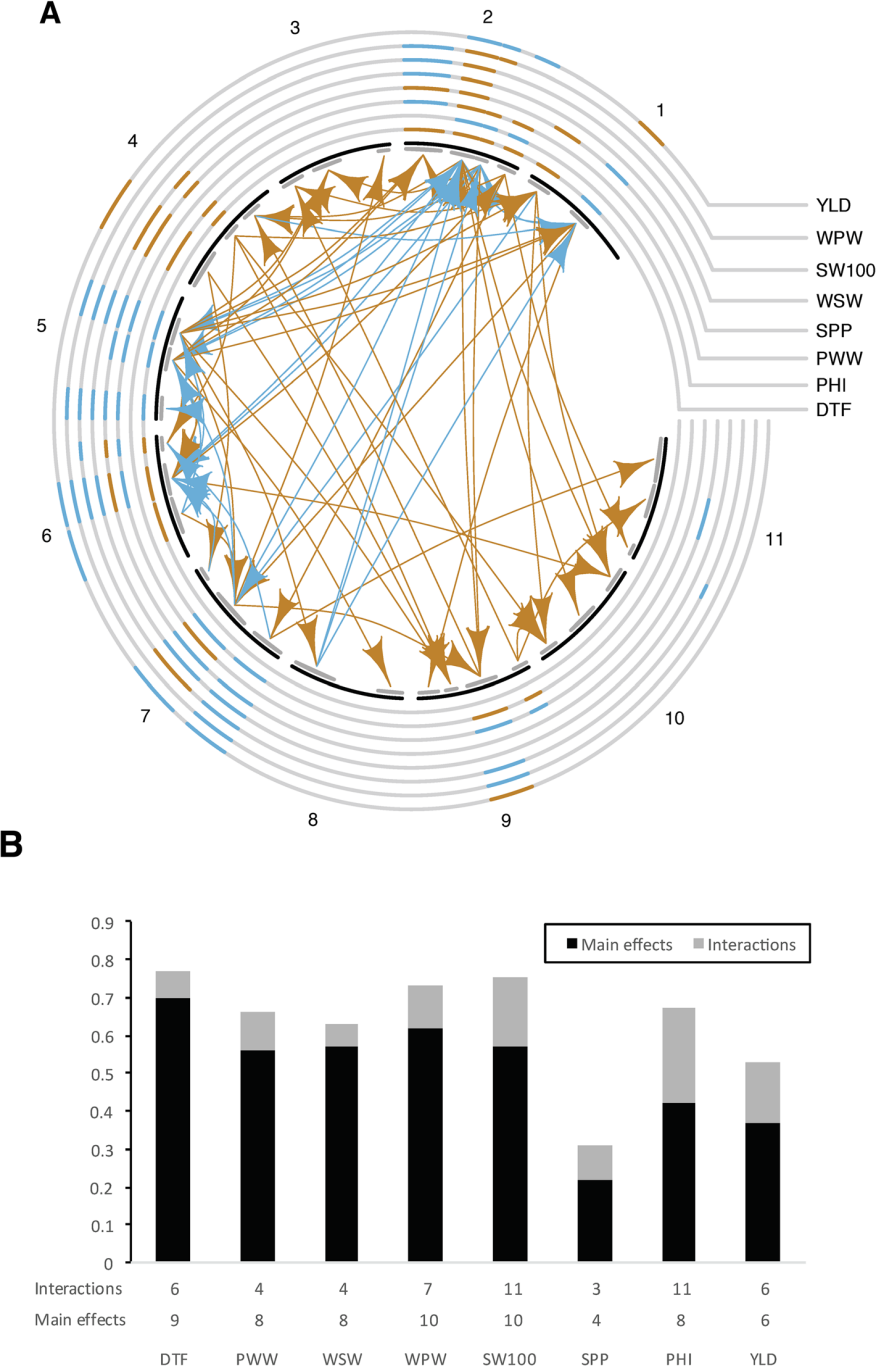
Network plot of pleiotropy and epistasis QTL interactions derived from CAPE. **a** The main effects are plotted in the circles with the ICA Bunsi positive allele in brown and SXB405 positive allele in blue. The interactions are shown in the arrows with enhancing (blue) and suppressing (brown) effect. DTF: Days to Flower; PHI: Pod Harvest Index; PWW: Pod Wall Weight; SPP: Seeds per Pod; SW100: One-hundred Seed Weight; WSW: Whole Seed Weight; WPW: Whole Pod Weight. **b** Variance explained by the main effects and the interactions, and the number of factors included in the final model

All nine QTLs for PHI clustered with other QTLs (Figs. 2 and 3). They grouped with WSW and WPW on Pv01, with PWW on Pv02, with WSW, SPP, YLD, and WPW on Pv04, with DTF, SPP and YLD on Pv05, with WSW, WPW, SW100, PWW, and YLD on Pv06, and with YLD and Pv07. For three of the four PHI QTLs clustering with YLD QTLs (Pv04, Pv05, and Pv07), SXB405 provided the positive allele for each of the QTLs. In contrast, the PHI QTL originated in the ICA Bunsi parent and the YLD QTL in the SXB405 parent.

### Genetic interactions

#### Epistatic interactions among QTLs for the same trait

Digenic epistatic interactions were analyzed for the average of the traits across environments (Table 5) and within each environment (Additional file 2: Table S2). Across environments, epistatic interactions were found for 13 pairwise interactions, including PWW, WSW, SW100, PHI, and YLD (Table 5). Six epistatic QTL combinations were detected for PHI, two for PWW, three for WSW, and one each for WPW, SW100, and YLD. Furthermore, we found 51 epistatic interactions when analyzing each environment separately (Additional file 2: Table S2). The interactions were more consistent across treatments than across years. When analyzing the overall mean, PHI had the largest number of epistatic interactions.

**Table 5:** Digenic-epistatic interactions across environments for pod wall weight (PWW), whole-seed weight (WSW), whole-pod weight (WPW), 100-seed weight (SW100), pod harvest index (PHI) and yield (YLD)

Trait	Position 1	Interval 1	Position 2	Interval 2	LOD	PVE	Add1	Add2	A x A
PWW	Pv07.94	ss715646018-ss715639206	Pv09.63	ss715646367-ss715647626	4.0	7.4	−0.004	−0.014	−0.010
PWW	Pv09.0	ss715641128-ss715647842	Pv11.28	ss715645480-ss715645542	3.9	2.6	0.002	−0.007	−0.009
WSW	Pv05.43	ss715648643-ss715649151	Pv06.39	ss715646419-ss715645797	3.7	14.4	−0.001	0.002	0.021
WSW	Pv01.39	ss715639556-ss715649290	Pv10.53	ss715645501-ss715647381	3.5	17.7	0.001	− 0.001	0.024
WSW	Pv03.58	ss715639345-ss715639245	Pv11.38	ss715646672-ss715650431	3.6	16.2	−0.001	0.002	0.023
WPW	Pv05.41	ss715640122-ss715640788	Pv07.46	ss715646455-ss715644972	4.2	36.3	−0.002	−0.003	0.031
SW100	Pv07.41	ss715646353-ss715649276	Pv09.62	ss715646367-ss715647626	3.8	1.3	0.028	−0.502	0.312
PHI	Pv01.87	ss715645248-ss715645244	Pv06.3	ss715641022-ss715648313	3.6	4.7	−0.001	0.001	0.004
PHI	Pv02.1	ss715646675-ss715649308	Pv06.29	ss715645033-ss715648132	3.9	3.6	0.000	0.001	−0.004
PHI	Pv02.37	ss715650549-ss715650341	Pv09.0	ss715641128-ss715647842	3.6	3.0	−0.001	0.000	−0.004
PHI	Pv06.17	ss715649329-ss715646829	Pv09.26	ss715647012-ss715639456	3.6	3.1	−0.001	0.000	−0.003
PHI	Pv06.30	ss715648132-ss715650285	Pv10.34	ss715645510-ss715645516	4.0	3.6	0.001	−0.001	0.004
YLD	Pv04.13	ss715646227-ss715646223	Pv05.87	ss715646707-ss715646695	4.4	5.6	29.85	−22.92	−61.79

#### Pleiotropy and epistasis across traits

We further modeled pleiotropy and epistasis jointly with CAPE (Tyler et al. 2013, 2016). Analyzing the mean phenotypes across environments, there were genomic areas that had a pleiotropic effect for all traits, especially in Pv02, Pv05, Pv06 and Pv07 (Fig. 3). In addition, we found 47 loci, which were interacting in a network involving all linkage groups. In all traits, the main effects explained most of the variance (Fig. 3). However, for SW100, PHI and YLD, the interactions accounted for 18, 25, and 16% of the variation, respectively.

## Discussion

This study had three major goals: 1) to estimate the differential effect of intermittent and terminal drought on yield, PHI, and its components; 2) to identify QTLs for these traits; and 3) to estimate the role of pleiotropy-tight linkage and epistasis in controlling PHI.

### Differential effect of drought type on yield, PHI, and its components

The full population was grown for two years (2013 and 2014); the first year, both a terminal drought and intermittent drought treatment were imposed, while in the second year, only a terminal drought was considered. The dry conditions of the field site in Davis, California, allowed us to control the drought level through irrigation as rainfall during the summer is very rare, localized, and limited. In general, for all the traits, we found a significant effect in the treatment effects within each year, indicating that the reduced number of irrigations had imposed a sufficient experimental drought stress.

While the drought stress under terminal drought was encountered after flowering, the intermittent drought treatment overlapped during mid flowering. Nevertheless, the difference between years was even more notable. Heat waves are an occasional issue and can induce inflorescence and pod abortion, as well as reduced fertilization [37–39]. There was a heat wave during flowering in the second year causing flower drop and a lower number of seeds per pod. Nevertheless, the yields recovered, probably through re-growth and compensation that can occur in indeterminate cultivars [38, 40]. We found a significant genotype-by-environment interaction in all traits; however, pod traits (PWW, WPW, WSW, and PHI), SW100, and DTF showed high broad-sense heritability, while, unsurprisingly, SPP and YLD showed lower heritability (Table 1).

When comparing the effects of intermittent and terminal drought to the control, PHI was similar in terminal drought and the control [29, 40], but was lower in intermittent drought. These results suggest that photosynthate allocation towards pods is enhanced under TD but decreased under ID. The SPP was lower in ID than in control and TD, probably because of heat stress during the reproductive stage that has an effect on fertilization and seed abortion [38, 41]. Therefore, photosynthate allocation could be hampered due to lower sink strength [14].

The study of correlated traits, which arise through pleiotropy and linkage disequilibrium [42], is important because they influence the progress from selection in breeding. Across environments, the correlations of YLD with the other traits were consistent. When comparing the mean of phenotypic traits across environments, PHI had the highest correlation with YLD (r = 0.5) among traits, suggesting the importance and usefulness of the trait for indirect selection for yield [6, 27]. Among its component traits, PHI had a higher absolute correlation with PWW (− 0.47) than with WSW (0.32) and SW100 (0.17), suggesting both the importance of photosynthate allocation to pods and its remobilization to the seeds. The number of seeds per pod was also correlated with PHI (0.41), suggesting that sink strength due to the number of seeds is higher than sink strength based on seed size.

Individual seed weight usually has lower plasticity and higher heritability [43, 44]. The number of seeds per pod is highly affected by temperature [37, 38, 45] and drought stress [46]. The latter is consistent with our findings that the number of seeds per pod was significantly lower in the second year, when the plants suffered a high heat episode during flowering and the differences between treatments were only significant under intermittent drought, when drought stress was present at the beginning of flowering. Interestingly, there was no correlation between SPP and PWW or SW100, in addition to the high heritability of the latter two traits, and medium correlation between pod wall and seed weight. This observation suggests that pod walls have relatively low plasticity; hence, seed and pod morphologies may be inherited differently.

### Identification of QTLs

#### Map development

A genetic map was developed with the BARCBean6K_3 SNP chip platform with an average density of 3 markers per cM. The marker density of linkage maps of other populations genotyped with the same BARCBean6K_3 SNP chip had an average of 1.61 and a range from 0.64 to 2.4 markers per cM [25, 29, 30, 47, 48]. Overall, the sample size and marker density for our analyses were adequate for QTL mapping [49, 50].

However, the distribution of SNPs was uneven over the eleven linkage groups. In most of the metacentric chromosomes ([51]: Pv02, Pv03, Pv04, Pv05, Pv07, Pv08, Pv10, and Pv11, the SNPs were distributed towards the ends of the two arms. An exception was Pv08, a metacentric chromosome, with a large region of towards one end of the chromosome. This large monomorphic area may be due to common ancestry from ICA Bunsi, as this cultivar was not only one of the parents of this RI population but has also been used as a parent of at least two of the lines (A686, SEA15) used in the development of the other parent, SXB405 (S. Beebe, unpublished information). It may also contain genes that were under the same selection during the development of the two RI parents. Traits that have been mapped to these regions include principally growth habit and phenology traits. These included number of nods and pods on the main stem, harvest index, and days to flowering and maturity [52], lodging and seed weight [53], and shoot ant total weight, hypocotyl length, root weight, and SPAD index (chlorophyll content [54]. This overall focus on plant type is also reflected in the use of ICA Bunsi as a parent to obtain white mold resistance. Miklas et al. [55] mapped several QTLs for white mold (*Sclerotinia sclerotiorum* Lib. de Bary) avoidance to this same region on Pv08, including canopy porosity, height, and lodging, suggesting that selection for a more upright and porous plant type is controlled at least partially by this region on Pv08 and has been selected by multiple breeding programs, whether directly for growth habit or for avoidance of white mold.

#### QTL mapping

Additive and epistatic marker-trait association analysis for PHI, its components, and YLD were performed for each irrigation treatment/year combination as well as for the overall mean across environments. In addition, we included a QTL by environment analysis to estimate the consistency of the marker QTLs across environments.

Nine QTLs were detected for PHI with the positive alleles of the QTLs contributed by both parents. A major and stable QTL was detected on linkage group Pv07 explaining 17% of the additive variation across environments. It was detected in all environments and had a low (3%) additive-by-environmental percentage of explained variation. SXB405 contributed the positive allele. No QTL in Pv07 has been reported before in previous analyses [28–30, 32].

The other eight QTL were not detected in all environments, but were detected mostly either in one year or the other.

We found eight QTLs for yield. None of the QTLs were detected in all environments; instead, they appeared in one year or the other. However, despite the inconsistency of yield QTLs, a frequent observation among QTL studies [32, 56], three QTLs were detected when mapping the mean yield across environments. The co-location of some yield and PHI QTLs across populations (and environments) is encouraging. Furthermore, the sign of the parental effect of all the QTLs across environments was similar, that is, the allele that had a positive effect in one environment did not have a negative effect in another environment.

### Role of pleiotropy-tight linkage and epistasis in controlling PHI

#### Clustering of QTLs and pleiotropy

Pleiotropy refers to the condition when one genetic element regulates more than one gene product and trait [57]. Pleiotropy can be assessed first by the clustering of genes controlling different traits to a specific sequence although co-location does not necessarily imply pleiotropy, as it could be due to tightly linked genes. The expectation of the underlying variation and genetic control of PHI would be that the photosynthate remobilization rate, as well the intrinsic allocation to pod walls and seeds co-localize. As expected, PHI QTLs co-localized with QTLs of some of its components. However, the components were co-localized differentially. Three QTLs co-localized solely with PWW, two with the seed and WPW, one with WSW and SPP, one with SPP and DTF, and one with pod wall, seed and whole pod weight and SW100. In addition, the major QTL at Pv07 did not co-localize with any of its components, but it co-localized with a yield QTL.

This confirms that the variation in PHI is not only a measure of photosynthate remobilization capacity, but also depends on the variation in allocation of photosynthates to seeds and pod-walls. For example, the seed weight in a pod is a function of the number of seeds and the individual seed weight. The former is a factor of the number of ovules and fertilization/abortion rates (Nakamura, 1986, 1988). The latter is a factor of seed volume and density. The seed volume in turn is controlled by seed dimension variation (length, width, thickness, shape, and distance between ovules within the pod). The weight of pod wall per pod would also be a factor of the pod dimension and wall thickness and density. All these traits can have unique or pleotropic genetic control as well as being constrained by the availability of photoassimilates, water, and nutrients [14, 58].

Four of the eight QTLs for yield co-located with other traits. Three of them co-located with PHI, underlying the importance of photosynthate allocation and remobilization for high productivity. No co-localization between QTLs for PHI with YLD has been observed before [28–30, 32].

#### Evidence of epistasis

A substantial number of digenic epistatic interactions were observed across and within environments. However, the interactions were not usually consistent among environments, especially between the two years. In addition, from the 13 pairwise interactions found in the mean across environments, only 5 were detected within years, confirming the complexity and lack of consistency of epistasis. Nevertheless, we found significant epistatic networks in all traits and linkage groups. For some traits, like PHI and yield, epistasis accounted for a large part of the variation. Our results support previous findings of digenic interactions for yield and yield component traits in beans, as well the importance of interacting genomic regions that do not show main effects [23, 34]. They also suggest the importance of higher-order epistasis [23]. Furthermore, epistasis, in addition to gene complementarity, would explain the significant transgressive segregation we found in all traits [59]. As epistatic effects are substantial, especially in complex traits like yield, genomic selection in beans would benefit from models that include epistasis, which have been proven useful in improving the accuracy in other species [60–64]. Accumulating the right epistatic combinations is complex because of the large number of factors and, thus, the low probability of recovery of progeny with the desired gene combinations. However, improved capabilities of high-throughput genotyping and phenotyping, allows us to relatively cheaply screen large numbers of recombinant individuals with a large number of markers.

## Conclusions

Through the detailed phenotypic and genetic evaluations under different irrigation regimes, we have identified a substantial number of genomic regions associated with photosynthate allocation and remobilization under drought stress in common bean. We found a significant effect of the water regime on all the traits, at both the phenotypic and QTL levels. However, the year effect was stronger. With some exceptions, when the QTL was not consistent across environments, they tended to be detected across treatments within the same year. We found several QTLs for PHI, including a major QTL on Pv07. In addition, QTLs for PHI co-localized with its component traits, but pod wall or seed allocation and remobilization patterns varied among QTLs. PHI co-localized with three out of seven QTLs for YLD, underscoring its importance as a determinant of productivity. All the QTLs had the same sign of effect between environments, strongly suggesting there are no tradeoffs among water regimes. We also found substantial evidence for epistasis, especially for yield and PHI. Although epistasis was not consistent across environments, it explained a considerable part of the phenotypic variation. Our results support an approach of joint genomic and phenotypic selection of yield and its components. PHI is a valuable selection goal in both well-watered and water-stressed environments because of its high correlation with yield and its higher heritability than yield. Information about physiological traits can be useful for improving the efficiency of selection and abiotic stress resilience [11, 65]. Ultimately, more robust beans, able to better withstand yield-reducing stresses, will enhance its nutritional role as a source of dietary protein, fibers, vitamins, and minerals.

## Methods

### Population development

To investigate the genetic basis of PHI [defined as seed dry weight per pod at harvest or whole-seed weight (WSW) over average dry pod weight, including seeds, at harvest or whole-pod weight (WPW)] and other yield-related traits, a bi-parental population was evaluated consisting of 226 F_9_ Recombinant Inbred Lines (RILs) from the cross of ICA Bunsi and SXB405, developed at the Centro Internacional de Agricultura Tropical (CIAT), Colombia. This population was selected for a QTL analysis because a previous study using a subset of this population (78 lines, F_4:6_) found large phenotypic variation and transgressive segregation for PHI, as well as relatively high heritability and correlation with yield [27]. This study provides a more extensive analysis on the entire population; it also combines phenotypic and genotypic data, leading to an as yet unperformed QTL analysis in this population.

ICA Bunsi is a navy type bean developed in 1968 by the Instituto Colombiano Agropecuario (ICA) from a cross between Magdalena 8 and Japón 3 [66]. It has been used extensively in Canadian breeding programs [67], due to its high productivity and resistance to white mold (*Sclerotinia sclerotiorum*) [68, 69]. ICA Bunsi carries the *I* gene, which confers Bean Common Mosaic Virus resistance [70]. SXB405 is a cream-colored type experimental line developed at CIAT from a four-way cross (A 686/A 774//NXB 80/SEA 15) and selected for its high productivity under drought and common bacterial blight (*Xanthomonas axonopodis* pv. *phaseoli*) resistance [27, 71]. Both genotypes belong to the Mesoamerican gene pool, are photoperiod-neutral, and have a type II (indeterminate, bush) growth habit [27].

### Trial design

**Fig. 4:**
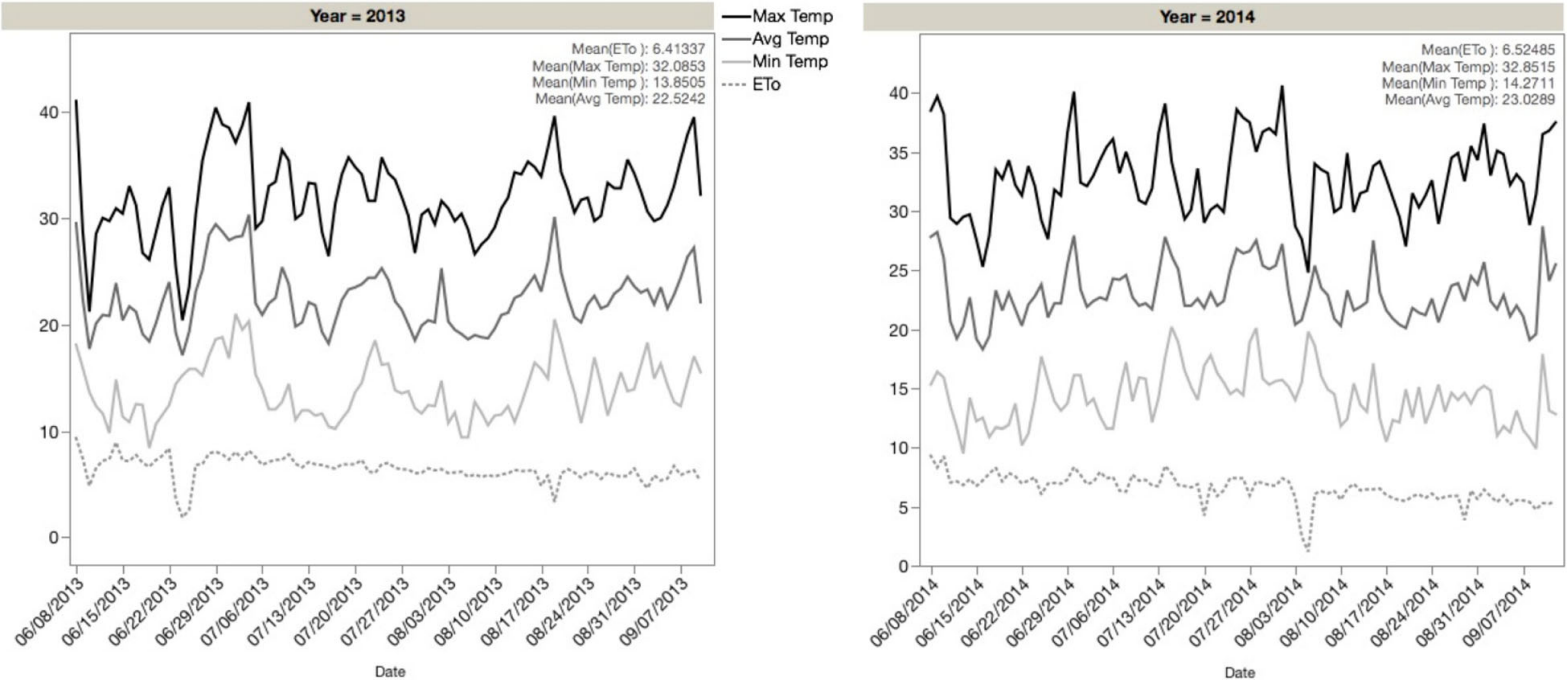
Field environmental conditions of the experiment at the Plant Sciences Farm, University of California, Davis in 2013 (A) and 2014 (B). Maximum, average, and minimum temperature (0C) and evapotranspiration (ETo, mm) were obtained from the Davis station of the California Irrigation Management Information System (CIMIS; www.cimis.water.ca.gov)

Field experiments were carried out at the Plant Sciences Farm at the University of California, Davis (38.53 °N, 121.78 °W) for a total of five environments in two years and three water regime treatments. The soil type of the site belongs to the Yolo series, a member of fine-silty loam, mixed, nonacid, thermic Mollic Xerofluvents, considered well-drained, with slow to medium runoff and moderate permeability (https://soilseries.sc.egov.usda.gov). The seeding was carried out on the 5th of June in 2013 and 8th of June in 2014. The plants were harvested on the 10th of September in 2013 and 12th of September in 2014. All water was provided by irrigation as there were no rain events during the experiments. In 2013, there were three irrigation treatments: terminal drought (TD), intermittent drought (ID), and full irrigation (well-watered). The full irrigation treatment received four irrigations while the intermittent drought received the first and third irrigation, and the terminal drought received the first and second irrigation. Only terminal drought and full irrigation treatments were carried out in 2014, receiving 4 irrigations for the full irrigation treatment, and the first two irrigations for terminal drought. The second irrigation of the terminal drought was applied during early flowering. The timing of the irrigation was decided according to weather and evapotranspiration (Fig. 4). The agricultural management was according to standard practices [72]. The parental lines and 226 of the RIL lines were planted in a split plot design with 3 blocks and one repetition per block. The experimental unit was a plot of 60 plants grown in two 3 m-long rows and 76 cm between rows (density of 131,578 plants per hectare).

### Phenotyping

From each plot, 15 to 20 pods were collected at random across the plot during harvest and dried for 5 days at 50 °C. Seed and pod number and pod mass were recorded. The seeds and pod walls were weighed separately. Pod harvest index was calculated as the proportion of seed biomass to the overall pod biomass (sum of pod wall + seed biomass) [17]. Days to flowering (DTF) was assessed when at least 50% of the plants in the plot had an open flower. Agronomic yield was weighed after four days of drying at 40 °C to standardize grain water content. Yield was not measured on the intermittent drought treatment in 2013 as the plots were mixed after cutting due to unexpectedly high winds.

### Statistical analyses

The data were analyzed in a linear mixed model: genotype, irrigation treatment and their interaction were considered as fixed effects, while the block and the interaction of block and treatment were considered as random effects. Each irrigation treatment and year was combined into one treatment with five levels. This approach was chosen since one irrigation pattern (intermittent drought) was not present in the second year. In addition, stress levels can vary between years, as weather variables and the precise timing of irrigation (in relation to growth and development of the plant) might have differential effects.

Statistical analyses were performed in the R environment [73], with the “lme4” package [74]. The “lmerTest” was used to determine the effect significance and least squared mean calculation [75], with type III hypothesis testing using the Satterthwaite approximation for degrees of freedom. R squared was obtained with the “piecewiseSEM” package [76]. The coefficient of variation was calculated with “sjstats” [77]. Broad sense heritability was estimated with REML [78] as ${h}^2={\sigma}_G^2/\left({\sigma}_G^2+{\sigma}_{GT}^2/e+{\sigma}_E^2/ er\right)$ where ${\sigma}_G^2$ is the genetic variance, ${\sigma}_{GT}^2$ is the variance of the genotype by treatment interaction, ${\sigma}_E^2$ is the variance of the experimental error, e is the number of environments and r the number of repetitions per environment. The correlation between traits was calculated and plotted with the “ggally”package [79].

### Genotyping

A single F_7_ plant of each of the 226 RILs and the two parents were used for extracting DNA with a modified ammonium acetate based protocol [80]. The samples were genotyped with 5398 SNP markers from the BARCBean6K_3 BeadChip SNP chip platform [81] at the USDA-ARS Soybean Genomics Improvement Laboratory, Beltsville, MD. After filtering, GenomeStudio Module v1.8.4 (Illumina Inc., San Diego, CA, USA) was used for automatic SNP calling. After subsequent manual adjustments, filtering for quality control, and a 0.15 Gencall score cutoff, 5186 SNPs remained.

### Genetic map construction

The SNP segregation database was filtered for marker monomorphism, missing data less than 5%, co-located markers, genetic clones and segregation distortion. The construction of the genetic linkage map markers was carried out with the “qtl” and “asMAP” packages using the Kosambi function [82, 83]. From 1039 polymorphic markers between the parents, three-hundred seventy-eight markers remained after filtering for missing data and co-located markers.

### QTL mapping

The identification of QTLs was performed using inclusive composite interval mapping (ICIM) with QTL IciMapping version 4.1 [84]. ICIM uses a two-step strategy using stepwise regression for marker selection followed by interval mapping using phenotypes adjusted from the markers selected in the first step except the flanking markers of the current marker interval [85, 86]. All the traits/environment combinations, as well as the mean value across environments, were analyzed with the ICIM-ADD for testing additive interactions. A probability of 0.001 and a step of 1 cM was used for the stepwise regression and the significance LOD threshold was calculated by 1000 permutations in each trait at a significance level of 0.01. QTL-by-environment interaction and stability was studied with the ICIM-QEI mapping function, which detects average and environment specific effects [87]. To study epistasis, two-dimensional scanning was carried out in each trait/environment combination by the ICIM-EPI method [84], with a 1 cM step and a minimum *p*-value and LOD score of 0.01 and 3.5, respectively.

Because of evidence of both pleiotropy and epistasis, we further modeled them through the “CAPE” package [88, 89]. CAPE infers epistatic networks of QTLs affecting one or more traits First, it integrates multiple related phenotypes as eigentraits through singular value decomposition to de-correlate traits [88, 90]. We used first three eigentraits, capturing 85% of the variance across traits. A single-variant scan was performed with linear regression with 1000 permutations to obtain the significance threshold. After selection of markers as covariates, a pairwise scan, for interaction detection, was carried out with 500,000 permutations to obtain empirical *p* values and a maximum Pearson correlation between markers of 0.7 to avoid testing highly correlated markers. The regression coefficients of single markers and marker interaction on the eigentraits were combined and reparameterized to detect between marker-pair influence independently of the eigentraits. Then, the influences were translated to the original phenotypes. The final network was obtained with a minimum adjusted p value of 0.01, and a minimum threshold power of 1. We estimated the variance explained by the QTLs as main effects and the interacting markers using linear regression. The Stepwise regression with model selection using the Akaike information criterion (AIC) in the package “MASS” [91] was used first and only the significant markers and interactions were included in the final model.

## Additional files

Additional file 1: **Table S1.** QTLs identified for various phenotypic traits in different years and under different water availability treatments. C: well-watered control; ID: intermittent drought; TD: terminal drought. PVE: proportion of variation explained by QTL. (XLSX 28 kb)

Additional file 2: **Table S2.** Digenic epistatic interactions in each environment (year, irrigation treatment). C: well-watered control; ID: intermittent drought; TD: terminal drought. (XLSX 15 kb)

## Abbreviations

AIC: Akaike information criterion
CAPE: Software package
cM: centiMorgan
DTF: Days to Flower
ID: Intermittent drought
LOD: Logarithm of the odds ration
PHI: Pod Harvest Index
PWW: Pod Wall Weight
QTL: Quantitative trait locus
RI: Recombinant inbred
RIL: Recombinant inbred line
SNP: Single-nucleotide polymorphism
SPP: Seeds per pod
SW100: One-hundred seed weight
TD: Terminal drought
WPW: Whole pod weight
WSW: Whole seed weight (average seed weight per pod)

## References

[1] Long SP, Zhu X-G, Naidu SL, Ort DR (2006). Can improvement in photosynthesis increase crop yields?. Plant Cell Environ.

[2] Zhu X-G, Long SP, Ort DR (2010). Improving photosynthetic efficiency for greater yield. Annu Rev Plant Biol.

[3] Long Stephen P, Marshall-Colon A, Zhu X-G (2015). Meeting the global food demand of the future by engineering crop photosynthesis and yield potential. Cell.

[4] Hay RKM (1995). Harvest index: a review of its use in plant breeding and crop physiology. Ann Appl Biol.

[5] Rosales-Serna R, Kohashi-Shibata J, Acosta-Gallegos JA, Trejo-López C, Ortiz-Cereceres JN, Kelly JD (2004). Biomass distribution, maturity acceleration and yield in drought-stressed common bean cultivars. Field Crops Res.

[6] Klaedtke SM, Cajiao C, Grajales M, Polanía J, Borrero G, Guerrero A, Rivera M, Rao I, Beebe SE, Léon J (2012). Photosynthate remobilization capacity from drought-adapted common bean (*Phaseolus vulgaris* L.) lines can improve yield potential of interspecific populations within the secondary gene pool. J Plant Breed Crop Sci.

[7] Polania JA, Poschenrieder C, Beebe S, Rao IM (2016). Effective use of water and increased dry matter partitioned to grain contribute to yield of common bean improved for drought resistance. Front Plant Sci.

[8] Medina V, Berny-Mier y Teran JC, Gepts P, Gilbert ME (2017). Low stomatal sensitivity to vapor pressure deficit in irrigated common, lima and tepary beans. Field Crop Res.

[9] Sponchiado BN, White JW, Castillo JA, Jones PG (1989). Root growth of four common bean cultivars in relation to drought tolerance in environments with contrasting soil types. Exp Agric.

[10] Polania J, Rao IM, Cajiao C, Grajales M, Rivera M, Velasquez F, Raatz B, Beebe SE (2017). Shoot and root traits contribute to drought resistance in recombinant inbred lines of MD 23–24 × SEA 5 of common bean. Front Plant Sci.

[11] Wallace DH, Baudoin JP, Beaver J, Coyne DP, Halseth DE, Masaya PN, Munger HM, Myers JR, Silbernagel M, Yourstone KS (1993). Improving efficiency of breeding for higher crop yield. Theor Appl Genet.

[12] Schneider KA, Brothers ME, Kelly JD (1997). Marker-assisted selection to improve drought resistance in common bean. Crop Sci.

[13] Collard BCY, Mackill DJ (2008). Marker-assisted selection: an approach for precision plant breeding in the twenty-first century. Philos Trans R Soc B Biol Sci.

[14] Tanaka A, Fujita K (1979). Photosynthesis and yield components in relation to grain yield of the field beans. J Faculty Agric Hokkaido Univ.

[15] Broughton WJ, Hernandez G, Blair M, Beebe S, Gepts P, Vanderleyden J (2003). Beans (*Phaseolus* spp.) - model food legumes. Plant Soil.

[16] Gepts P, Aragão FJL, Ed B, Blair MW, Brondani R, Broughton W, Galasso I, Hernández G, Kami J, Lariguet P, Moore PH, Ming R (2008). Genomics of *Phaseolus* beans, a major source of dietary protein and micronutrients in the Tropics. Genomics of Tropical Crop Plants.

[17] Beebe SE, Rao IM, Blair MW, Acosta-Gallegos JA (2013). Phenotyping common beans for adaptation to drought. Front Physiol.

[18] Ramirez-Cabral NYZ, Kumar L, Taylor S (2016). Crop niche modeling projects major shifts in common bean growing areas. Agric For Meteorol.

[19] RAO I. M., BEEBE S. E., POLANIA J., GRAJALES M., CAJIAO C., RICAURTE J., GARCÍA R., RIVERA M. (2016). Evidence for genotypic differences among elite lines of common bean in the ability to remobilize photosynthate to increase yield under drought. The Journal of Agricultural Science.

[20] Prudhomme C, Giuntoli I, Robinson EL, Clark DB, Arnell NW, Dankers R, Fekete BM, Franssen W, Gerten D, Gosling SN (2014). Hydrological droughts in the 21st century, hotspots and uncertainties from a global multimodel ensemble experiment. Proc Natl Acad Sci.

[21] Kissoudis C, van de Wiel C, Visser RGF, van der Linden G (2016). Future-proof crops: challenges and strategies for climate resilience improvement. Curr Opin Plant Biol.

[22] Jez JM, Lee SG, Sherp AM (2016). The next green movement: plant biology for the environment and sustainability. Science.

[23] Johnson WC, Gepts P (2002). The role of epistasis in controlling seed yield and other agronomic traits in an Andean x Mesoamerican cross of common bean (*Phaseolus vulgaris* L.). Euphytica.

[24] Blair MW, Iriarte G, Beebe S (2006). QTL analysis of yield traits in an advanced backcross population derived from a cultivated Andean x wild common bean (*Phaseolus vulgaris* L.) cross. Theor Appl Genet.

[25] Hoyos-Villegas V, Song Q, Wright EM, Beebe SE, Kelly JD (2016). Joint linkage QTL mapping for yield and agronomic traits in a composite map of three common bean RIL populations. Crop Sci.

[26] Beebe Steven (2012). Common Bean Breeding in the Tropics. Plant Breeding Reviews.

[27] Assefa T, Beebe SE, Rao IM, Cuasquer JB, Duque MC, Rivera M, Battisti A, Lucchin M (2013). Pod harvest index as a selection criterion to improve drought resistance in white pea bean. Field Crop Res.

[28] Asfaw A, Blair MW, Struik PC (2012). Multienvironment quantitative trait loci analysis for photosynthate acquisition, accumulation, and remobilization traits in common bean under drought stress. Genes Genomes Genetics.

[29] Mukeshimana G, Butare L, Cregan PB, Blair MW, Kelly JD (2014). Quantitative trait loci associated with drought tolerance in common bean. Crop Sci.

[30] Trapp JJ, Urrea CA, Cregan PB, Miklas PN (2015). Quantitative trait loci for yield under multiple stress and drought conditions in a dry bean population. Crop Sci.

[31] Kamfwa K, Cichy K, Kelly J (2015). Genome-wide association analysis of symbiotic nitrogen fixation in common bean. Theor Appl Genet.

[32] Diaz LM, Ricaurte J, Cajiao C, Galeano CH, Rao I, Beebe S, Raatz B (2017). Phenotypic evaluation and QTL analysis of yield and symbiotic nitrogen fixation in a common bean population grown with two levels of phosphorus supply. Mol Breed.

[33] Yuste-Lisbona F, González A, Capel C, García-Alcázar M, Capel J, Ron A, Santalla M, Lozano R (2014). Genetic variation underlying pod size and color traits of common bean depends on quantitative trait loci with epistatic effects. Mol Breed.

[34] Yuste-Lisbona FJ, González AM, Capel C, García-Alcázar M, Capel J, Ron AM, Lozano R, Santalla M (2014). Genetic analysis of single-locus and epistatic QTLs for seed traits in an adapted × nuña RIL population of common bean (*Phaseolus vulgaris* L.). Theor Appl Genet.

[35] Schmutz J, McClean P, Mamidi S, Wu G, Cannon S, Grimwood J, Jenkins J, Shu S, Song Q, Chavarro C (2014). A reference genome for common bean and genome-wide analysis of dual domestications. Nat Genet.

[36] Voorrips RE (2002). MapChart: software for the graphical presentation of linkage maps and QTLs. J Hered.

[37] Konsens I, Ofir M, Kigel J (1991). The effect of temperature on the production and abscission of flowers and pods in snap bean (*Phaseolus vulgaris* L.). Ann Bot.

[38] Shonnard GC, Gepts P (1994). Genetics of heat tolerance during reproductive development in common bean. Crop Sci.

[39] McClean PE, Burridge J, Beebe S, Rao IM, Porch TG (2011). Crop improvement in the era of climate change: an integrated, multi-disciplinary approach for common bean (Phaseolus vulgaris). Funct Plant Biol.

[40] Acosta Gallegos JA, Shibata JK (1989). Effect of water stress on growth and yield of indeterminate dry-bean (*Phaseolus vulgaris*) cultivars. Field Crop Res.

[41] Gross Y, Kigel J (1994). Differential sensitivity to high temperature of stages in the reproductive development of common bean (*Phaseolus vulgaris* L.). Field Crop Res.

[42] Saltz JB, Hessel FC, Kelly MW (2017). Trait correlations in the genomics era. Trends Ecol Evol.

[43] Motto M, Soressi GP, Salamini F (1978). Seed size inheritance in a cross between wild and cultivated common beans (*Phaseolus vulgaris* L.). Genetica.

[44] Sadras VO (2007). Evolutionary aspects of the trade-off between seed size and number in crops. Field Crop Res.

[45] Prasad PVV, Boote KJ, Allen LH, Thomas JMG (2002). Effects of elevated temperature and carbon dioxide on seed-set and yield of kidney bean (Phaseolus vulgaris L.). Glob Chang Biol.

[46] Ramirez-Vallejo P, Kelly J (1998). Traits related to drought resistance in common bean. Euphytica.

[47] Brisco EI, Porch TG, Cregan PB, Kelly JD (2014). Quantitative trait loci associated with resistance to Empoasca in common bean. Crop Sci.

[48] Heilig JA, Beaver JS, Wright EM, Song Q, Kelly JD (2017). QTL analysis of symbiotic nitrogen fixation in a black bean population. Crop Sci.

[49] Darvasi A, Weinreb A, Minke V, Weller JI, Soller M (1993). Detecting marker-QTL linkage and estimating QTL gene effect and map location using a saturated genetic map. Genetics.

[50] Li H, Hearne S, Bänziger M, Li Z, Wang J (2010). Statistical properties of QTL linkage mapping in biparental genetic populations. Heredity.

[51] Fonsêca A, Ferreira J, dos Santos T, Mosiolek M, Bellucci E, Kami J, Gepts P, Geffroy V, Schweizer D, dos Santos K (2010). Cytogenetic map of common bean (*Phaseolus vulgaris* L.). Chromosom Res.

[52] Koinange EMK, Singh SP, Gepts P (1996). Genetic control of the domestication syndrome in common-bean. Crop Sci.

[53] Moghaddam Samira Mafi, Mamidi Sujan, Osorno Juan M., Lee Rian, Brick Mark, Kelly James, Miklas Phillip, Urrea Carlos, Song Qijian, Cregan Perry, Grimwood Jane, Schmutz Jeremy, McClean Phillip E. (2016). Genome-Wide Association Study Identifies Candidate Loci Underlying Agronomic Traits in a Middle American Diversity Panel of Common Bean. The Plant Genome.

[54] Soltani A, MafiMoghaddam S, Oladzad-Abbasabadi A, Walter K, Kearns PJ, Vasquez-Guzman J, Mamidi S, Lee R, Shade AL, Jacobs JL (2018). Genetic analysis of flooding tolerance in an Andean diversity panel of dry bean (*Phaseolus vulgaris* L.). Front Plant Sci.

[55] Miklas PN, Porter LD, Kelly JD, Myers JR (2013). Characterization of white mold disease avoidance in common bean. Eur J Plant Pathol.

[56] Bernardo R (2016). Bandwagons I, too, have known. Theor Appl Genet.

[57] Brenner S, Miller J. Brenner's encyclopedia of genetics, 2nd edition. San Diego: Academic Press; 2013.

[58] Patrick JW, Colyvas K (2014). Crop yield components – photoassimilate supply- or utilisation limited-organ development?. Funct Plant Biol.

[59] Rieseberg LH, Archer MA, Wayne RK (1999). Transgressive segregation, adaptation and speciation. Heredity.

[60] Dudley JW, Johnson GR (2009). Epistatic models improve prediction of performance in corn. Crop Sci.

[61] Hu Z, Li Y, Song X, Han Y, Cai X, Xu S, Li W (2011). Genomic value prediction for quantitative traits under the epistatic model. BMC Genet.

[62] Wang D, Salah El-Basyoni I, Baenziger SP, Crossa J, Eskridge KM, Dweikat I (2012). Prediction of genetic values of quantitative traits with epistatic effects in plant breeding populations. Heredity.

[63] Howard R, Carriquiry AL, Beavis WD (2014). Parametric and nonparametric statistical methods for genomic selection of traits with additive and epistatic genetic architectures. G3: Genes Genomes Genetics.

[64] Akdemir D, Jannink J-L, Isidro-Sánchez J (2017). Locally epistatic models for genome-wide prediction and association by importance sampling. Genet Sel Evol.

[65] Ishitani M, Rao I, Wenzl P, Beebe S, Tohme J (2004). Integration of genomics approach with traditional breeding towards improving abiotic stress adaptation: drought and aluminum toxicity as case studies. Field Crop Res.

[66] Voysest VO (2000). Mejoramiento genético del frijol: legado de variedades de América Latina 1930–1999 [Genetic improvement of bean: legacy of varieties of Latin America 1930–1999].

[67] Navabi A, Balasubramanian P, Pauls KP, Bett K, Hou A (2014). Genetic diversity of the Canadian dry bean varieties released since 1930: a pedigree analysis. Crop Sci.

[68] Tu JC, Beversdorf WD (1982). Tolerance to white mold (*Sclerotinia sclerotiorum* (lib.) de bary) in ex Rico 23, a cultivar of white bean(*Phaseolus vulgaris* l.). Can J Plant Sci.

[69] Miklas PN, Hauf DC, Henson RA, Grafton KF (2004). Inheritance of ICA Bunsi-derived resistance to white mold in a navy × pinto bean cross. Crop Sci.

[70] Wehner TC (1999). Vegetable cultivar descriptions for North America. List 24. HortScience 1999.

[71] Beebe SE, Rao IM, Cajiao C, Grajales M (2008). Selection for drought resistance in common bean also improves yield in phosphorus limited and favorable environments. Crop Sci.

[72] Common dry bean production in California, second edition. UC ANR Publication. 8402.

[73] R Development Core Team (2017). R: A language and environment for statistical computing.

[74] Bates D, Mächler M, Bolker B, Walker S (2015). Fitting linear mixed-effects models using lme4. J Stat Softw.

[75] Kuznetsova A, Brockhoff PB, Christensen RHB. lmerTest Package: Tests in Linear Mixed Effects Models. J Stat Softw. 2017;82(13):1–26.

[76] Nakagawa S, Schielzeth H (2013). A general and simple method for obtaining R2 from generalized linear mixed-effects models. Methods Ecol Evol.

[77] Lüdecke D. sjstats: Statistical Functions for Regression Models. 2016. Available at https://CRAN.R-project.org/package=sjstats.

[78] Holland JB, Nyquist WE, Cervantes-Martínez CT. Estimating and interpreting heritability for plant breeding: an update. Plant Breeding Reviews. 2003;22:9–112.

[79] GGally: extension to ‘ggplot2’. R package version 1.3.1. https://cran.r-project.org/web/packages/GGally/index.html.

[80] Pallotta MA, Warner P, Fox RL, Kuchel H, Jefferies SJ, Langridge P. Marker assisted wheat breeding in the southern region of Australia. In: Proceedings of the 10th International Wheat Genetics Symposium: 1-6 Sept. 2003 2003. Paestum: Istituto Sperimentale per la Cerealicoltura, Rome, Italy. p. 789–91.

[81] Song Q, Jia G, Hyten DL, Jenkins J, Hwang E-Y, Schroeder SG, Osorno JM, Schmutz J, Jackson SA, McClean PE (2015). SNP assay development for linkage map construction, anchoring whole-genome sequence, and other genetic and genomic applications in common bean. G3: Genes Genomes Genetics.

[82] Wu H, Churchill GA, Broman KW, Sen S (2003). R/qtl: QTL mapping in experimental crosses. Bioinformatics.

[83] Taylor J, Butler D (2017). R package ASMap: efficient genetic linkage map construction and diagnosis. J Stat Softw.

[84] Meng L, Li H, Zhang L, Wang J (2015). QTL IciMapping: integrated software for genetic linkage map construction and quantitative trait locus mapping in biparental populations. The Crop Journal.

[85] Li H, Ye G, Wang J (2007). A modified algorithm for the improvement of composite interval mapping. Genetics.

[86] Wang J, Li H, Zhang L, Meng L (2016). User’s manual of QTL IciMapping ver.4.1.Mexico.

[87] Li S, Wang J, Zhang L (2015). Inclusive composite interval mapping of QTL by environment interactions in biparental populations. PLoS One.

[88] Tyler AL, Lu W, Hendrick JJ, Philip VM, Carter GW (2013). CAPE: an R package for combined analysis of pleiotropy and epistasis. PLoS Comput Biol.

[89] cape: Combined Analysis of Pleiotropy and Epistasis. Available at https://cran.r-project.org/package=cape.

[90] Tyler AL, Ji B, Gatti DM, Munger SC, Churchill GA, Svenson KL, Carter GW (2017). Epistatic networks jointly influence phenotypes related to metabolic disease and gene expression in diversity outbred mice. Genetics.

[91] Venables WN, Ripley BD (2002). Modern applied statistics with S.

